# Patient involvement in the development of a handbook for moderate rheumatoid arthritis

**DOI:** 10.1111/hex.12457

**Published:** 2016-04-18

**Authors:** Louise Prothero, Sofia Georgopoulou, Savia de Souza, Ailsa Bosworth, Lindsay Bearne, Heidi Lempp

**Affiliations:** ^1^ Academic Department of RheumatologyClinical Trials Group, King's College LondonFaculty of Life Sciences and MedicineWeston Education CentreLondonUK; ^2^ National Rheumatoid Arthritis SocietyMaidenheadBerkshireUK; ^3^ Academic Department of PhysiotherapyDivision of Health and Social Care ResearchKing's College LondonLondonUK

**Keywords:** intensive management, patient education, rheumatoid arthritis, self‐management, user involvement

## Abstract

**Background:**

Self‐management is a key recommendation for people with rheumatoid arthritis (RA). Educational materials may support self‐management, and increasingly patients are becoming involved with the development of these materials. The TITRATE trial compares the effectiveness of intensive management to standard care in patients with moderate RA across England. As part of the intensive management intervention, participants are given a handbook.

**Aim and objectives:**

The aim of this study was to develop a handbook to support the intensive management. The objectives were to: (i) involve patients in the identification of relevant information for inclusion in the TITRATE handbook; (ii) ensure the content of the handbook is acceptable and accessible.

**Design:**

We held an audio‐taped workshop with RA patients. The transcript of the workshop was analysed using thematic content analysis.

**Results:**

Five main themes were identified as follows: ‘rheumatoid arthritis treatment, perceptions of rheumatoid arthritis, the importance of individualized goals, benefits of self‐management and the patient handbook’. Feedback from the workshop was incorporated into the handbook, and patients’ anonymous testimonies were added.

**Conclusion:**

This study demonstrates that patient contribution to the development of educational material to support intensive management of RA is both feasible and valuable. A qualitative evaluation of the use and impact of the handbook with patients and practitioners is planned on completion of the TITRATE trial.

## Introduction

Rheumatoid arthritis (RA) is a long‐term, progressive, auto‐immune disease that causes joint destruction, pain, disability and reduced quality of life.^1^ RA is estimated to cost the UK economy in the region of £5 billion per year, through direct costs to the NHS and associated health‐care providers, and indirect costs associated with early mortality and loss of productivity.^2^ The precise aetiology of RA remains unclear; however, there is a wide array of known risk factors (e.g. smoking) for developing RA, approximately 50% of which are genetic.^1^

The importance of self‐management for the regulation of long‐term conditions has been widely recognized.^3^ Patient education is incorporated into most RA self‐management programmes, for example the Arthritis Self‐Management Programme,^4^ and the RA Self‐Management Programme developed by The National Rheumatoid Arthritis Society (NRAS) and Self‐Management UK (http://www.nras.org.uk/ra-self-management-programme).

Traditionally, patient education was generated by clinicians and researchers primarily based on a biomedical model.^5^ Its main purpose was the provision of disease‐related information. Recently, there has been a shift towards empowerment to address patient autonomy and the right and responsibility of patients to access health information and to engage in health‐related decisions.^6^, ^7^ Patient education, which aims to empower patients, may include information about treatment options and effective ways of managing their condition. Importantly, the content needs to be relevant and evidence‐based in a form that is acceptable and useful to patients.^8^ The improvement of patients’ understanding of treatment options may help to facilitate shared decision making between the patient and practitioner, and patients who are active participants in managing their health have better outcomes than those who are passive recipients of care.^9^

Increasingly, patients are involved in the development of health‐care services reflected by the popularity of approaches such as co‐design.^10^ Co‐design is based on direct face‐to‐face user and provider collaboration to develop products or services. It places ‘the experience goals of patients and users at the centre of the design process’.^11^ ^(p308)^ A Cochrane review was conducted on involving patients in the planning and development of health care.^11^ The authors found that the most frequently reported outcome was the production of either new or improved sources of information for patients. A more recent systematic review examined the effects of user involvement in developing patient information material.^12^ The review found that trials of patient information leaflets developed with user involvement were more detailed, readable, and had improved layout and illustrations compared to leaflets developed by health‐care professionals alone.

Patients with a variety of conditions, for example prostate cancer, irritable bowel syndrome and osteoarthritis, have been involved in developing patient materials.^13^, ^14^, ^15^ These have included information presented to patients during recruitment appointments and patient guidebooks. An evaluation of the osteoarthritis guidebook, after it had been used as part of a randomized controlled trial (RCT), found that it was acceptable and useful for both patients and health‐care professionals.^16^ To date, however, there have been no published reports of people with RA being involved in the development of materials to support the intensive management of their condition.

The current Treatment Intensities and Targets in Rheumatoid Arthritis Therapy (TITRATE) trial is a pragmatic RCT, which will compare the effectiveness of intensive management to standard care in RA patients with moderate disease activity across England.^17^ Intensive management is a complex intervention and consists of several components, including increased medication (subject to disease activity), psychosocial support and the provision of information. Trained rheumatology practitioners will provide participants with treatment support based on motivational interviewing techniques to help them to set goals and work on areas where they express difficulties, for example adherence to medication, pain, fatigue, exercise and smoking.^18^ At the same time, participants will be given a handbook that describes the components of intensive management. The handbook will also provide information on complementary management strategies such as coping with fatigue and managing stress.

The aim of this study was to develop a handbook to support the intensive management. Against this background of increased patient involvement in the design of educational materials for long‐term conditions, the objectives are to: (i) involve patients in the identification of relevant information for inclusion in the TITRATE handbook; (ii) ensure the content of the handbook is acceptable and accessible.

## Methods

**Figure 1:**
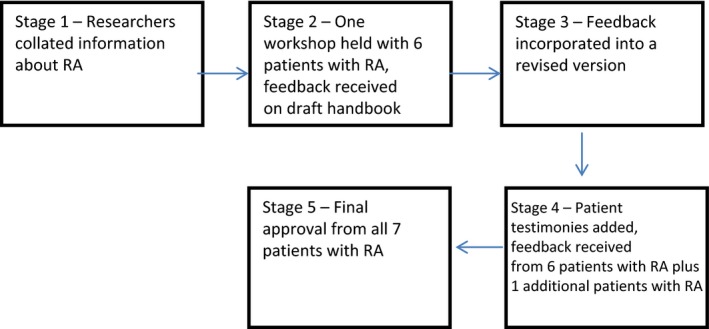
Development process of the Handbook (based on Plan, Do, Study, Act framework).^19^

The development of the patient handbook took place between May 2013 and April 2014, it involved five stages (see Fig. 1).

1. Firstly, two researchers (S.G., L.P.) collected information, for example current treatments for RA, intensive management in TITRATE, managing life with RA. The information was gathered from evidence‐based sources, for example publications and current clinical guidelines, expert medical and allied health practitioners, online from national charities NRAS and Arthritis Research UK (ARUK). It was then collated by one of the researchers (S.G.) into a draft handbook across nine chapters.
2. One patient workshop was organized at an inner city NHS Foundation Trust in June 2013. The workshop did not require ethical approval as its purpose was categorized as service development. The inclusion criteria comprised adults with a confirmed diagnosis of RA > 18 years, who were able to understand and communicate sufficiently in English to participate. All patients who agreed to take part were sent a participant information sheet describing the purpose of the workshop and its planned procedure. A week prior to the workshop, patients were sent a copy of the draft handbook. They were asked to read and consider the content and layout of the handbook and encouraged to make notes of any initial thoughts or feedback for the meeting.

The researchers who collected the information (S.G., L.P.) co‐facilitated the workshop which was conducted in a private room. Participants were asked to sign a written consent form before the start of the workshop. The consent form informed participants that taking part was voluntary, and withdrawal from the workshop would not affect the care they receive from the outpatient clinic. It also stated that the workshop was being audio recorded and anonymized quotations from the recording would be used in the future. The workshop began with a brief presentation and explanation about the purpose of the handbook within the context of the TITRATE trial. The draft handbook chapters were used to structure the remainder of the workshop. Taking each chapter in turn, the group was invited to provide feedback on all aspects of the draft document. There was a specific focus on the chapters about ‘intensive treatment for RA’ and ‘psychosocial support’ because these are key aspects of the intensive management intervention. The lead author (L.P.) took field notes during the workshop. The audio recording was transcribed verbatim, and the transcript and notes from the workshop analysed (L.P.) applying thematic content analysis.^20^ This involves ‘identifying, analysing, and reporting patterns (themes) within data’.^20^ ^(p79)^ The researcher referred back to the original transcript throughout the analysis to confirm participants’ accounts were presented accurately.

Using the audio recording and notes written during the workshop, feedback from participants relating to the content and layout was incorporated into a revised version of the handbook.
Following participants’ suggestions, selected anonymous testimonies that were expressed during the workshop were included to add context and personalize the content of the handbook. A further round of comments was then arranged for all workshop participants, and an additional patient who was unable to attend the workshop. Participants were sent the revised version of the handbook via email and asked to make any additional comments and send them to the researchers.
A final version of the handbook was sent to all who contributed to the development for approval prior to printing.

The multidisciplinary TITRATE team and NRAS also reviewed and provided feedback on the draft handbook before it was finalized for use in the TITRATE trial; however, these comments have not been included in this study.

## Results

The seven participants (six participants from the workshop plus one participant who provided separate feedback) were all female, aged 35–75 years (mean 60 years). Five participants were White British, one British Indian and one Black British. Participants were patients from one inner city hospital trust and NRAS patient volunteers**.** The workshop lasted approximately 75 min. Emergent themes were combined and refined, resulting in five main themes (see Table 1).

**Table 1:** Emergent themes and sub‐themes

Themes	Sub‐themes
1. Rheumatoid Arthritis treatment	Treatment decision making Information about intensive management
2. Perceptions of Rheumatoid Arthritis	Representation of RA as an ‘old person's disease’ Friends and family
3. The Importance of Setting Individualised Goals	
4. Benefits of Self‐Management	Self‐monitoring Pacing
5. The Patient Handbook	Patient testimonies Usability of Patient Handbook

### Theme 1. Rheumatoid arthritis treatment

#### Treatment decision making

The group discussed how they would like more information when deciding whether they should take a particular drug treatment for their RA. This included aspects such as the level of monitoring required, the method of administration and possible side‐effects. They also said they would like this information conveyed to them by their doctors in ‘clear’ language.

We are guided by our doctors but we need to be given as many facts as we can in a language that we understand […] I think it's things like “which medication” and “what that means”. So if you take this [medication] you're going to have to have this much monitoring. (Patient 2, 47 years)

Participants advocated that it would be beneficial to have time to go home and consider the information provided about a new treatment first, before making a decision, particularly if feeling unwell at the time of the appointment.

I went to sign the consent form to have it [suggested treatment] and then they [clinical team] told me the worst thing that could happen, and I went “I'm going to have to wait”. I went home and I thought about it [for a week] and read up online, had a chat to my children. (Patient 3, 66 years)

### Information about intensive management

The group had no knowledge of intensive management prior to reading the handbook. They thought the most important points to convey in the handbook were the aims of intensive management, benefits of intensive management above standard care and why it is important for patients to participate in the trial.

[…] the most crucial thing in that chapter is to understand or get across, what is the aim? Why is this important? […] So you need people to understand why you are suggesting this as an option. (Patient 2, 47 years)

Participants recommended including as much information as possible about the practical implications of taking part in the trial, in particular the frequency of monitoring that would be required.

The people who are going to be in the intensive treatment [arm], it will be very specific what they're going to have to do […] I think that needs to be in there [handbook] […] you will be doing x, y, z. Because then they know. (Patient 5, 62 years)

The group discussed the potential difficulties of regular monitoring that commonly includes travel to and from appointments and absence from work. This was an issue which was particularly salient to one of the patients who travels a long distance for her blood tests.

[…] this is my first time coming from East London […] this is something that I have to have a discussion with my doctor about, because I am going to be over there [East London] for a while. I don't know how this is going to be affecting my life. (Patient 6, 75 years)

### Theme 2. Perceptions of rheumatoid arthritis

#### Representation of RA as an ‘old person's disease’

Several participants in the workshop stated that RA is often confused with osteoarthritis and is commonly considered an ‘old person's disease’. They expressed frustration over other people's lack of knowledge about the variation in age of onset of RA. Some reported that they therefore prefer to use the term ‘autoimmune disease’ instead of RA when talking to others.

When people say to me, what have you got? I say, an auto‐immune condition ‐ because as soon as you say arthritis, anything to do with arthritis, there's an assumption that you'll get over it or take a pill and you'll be fine. (Patient 5, 62 years)

One participant did not like a picture of an old lady inserted in the handbook and suggested instead pictures of people spanning a wider age range.

One thing I didn't like was, I didn't like the picture of that woman. [Laughs] I thought she was too old […] I would have put her as osteoarthritis rather than rheumatoid. (Patient 4, 65 years)

### Friends and family

RA was described as ‘a lonely disease’ as often there are no visible signs, particularly early in the disease.

I think rheumatoid arthritis is quite a lonely disease, because you look quite “normal” and I have actually given up explaining to people that I don't feel well. (Patient 4, 65 years)

There is no “family” for RA sufferers. As it is a chronic disease, having a familiar receptionist who knows you and is pleased to see you, and nurses or consultants is vital for peace of mind. (Patient 7, 69 years)

The group discussed how RA can be difficult for partners, friends and family to understand. They considered the fluctuating nature of RA to be part of this problem because ‘some days you can do something and another day you might not be able to do the same thing’. Several drew from their own experiences of friends and family not being appreciative about their fatigue.

Then they go, “oh for goodness sake, go and sit down for half an hour. You'll be fine”. You're not putting it on. You won't sit down for five minutes and you'll be fine… (Patient 3, 66 years)

I find particularly if I go out with my friends […] because I seem quite “normal” when I'm out, but they don't realise that the rest of the time I'm actually at home catching up on sleep and everything. (Patient 1, 35 years)

Patients suggested including a section on friends and family in the patient handbook and put forward that the final document could be a useful additional resource for friends and family to learn more about RA.

[…] maybe show [the handbook] to your friends or relatives who don't know what RA is, then it's a nice thing to show I think. (Patient 1, 35 years)

It is also a very good document for family members to read. One of my granddaughters has been diagnosed with type 1 diabetes and I have been given an excellent booklet for me to keep – very useful and reassuring for all the family. (Patient 7, 69 years)

### Theme 3. The importance of setting individualised goals

Members of the workshop recommended that goals should be individualized, flexible and adaptive. They stressed the importance of an individualized treatment approach because people experience RA in different ways.

[The experience of the illness] it's a very individual thing and also depends upon the type of rheumatoid arthritis you have got. I was not damaged terribly badly in my joints, but suffer from chronic fatigue. (Patient 5, 62 years)

It was suggested that personal treatment goals should accommodate the fluctuating nature of RA and to avoid the potentially negative impact of unachievable goals.

The fear is when you are given a goal set by someone else, if you don't achieve it you feel very negative. And actually because the disease fluctuates the goal may be realistic at one point and then you have a big flare. (Patient 2, 47 years)

Diaries for activity scheduling were considered to be more useful compared to a reflective diary or journal. Several individuals in the group anticipated that a reflective diary would result in patients becoming too focused on their own symptoms and that it would ‘become negative as opposed to positive’.

### Theme 4. Benefits of self‐management

#### Self‐monitoring

The self‐management chapter which includes information about pacing, managing stress and coping with fatigue was well received by the group who considered these components as important to self‐monitor both their physical functioning and emotional status.

[…] with the breathing exercises and all that, it's stuff that's taken me four years to learn. I've only recently started doing like yoga and breathing. I wish I'd known about these things early on. (Patient 1, 35 years)

In a way you've got to develop a “self‐thing” about listening to your body ‐ because we're all different. It's like learning how to listen to your own signals, I suppose. (Patient 3, 66 years)

They emphasized the value of self‐monitoring, particularly to assess disease and functional status against their individual ‘normal’ state of RA. The benefits of self‐monitoring cited by patients included being able to recognize their limitations and identify if they are ‘slipping back’. One participant in the group pointed out the importance of learning to distinguish when symptoms are caused by the RA and when they caused by something else. She used the example of low mood and interpreting this as being caused by RA when it could be caused by a psychological illness such as depression.

So sometimes you're getting lower and it's maybe because of the disease. It may be for completely different reasons. But I think it's very easy to interpret it as being rheumatoid because rheumatoid can make you feel lousy. (Patient 2, 47 years)

#### Pacing

The group also reflected on the benefits of pacing, including being able to continue with activities such as employment and, therefore, avoid withdrawing from life and social isolation. They also discussed psychological advantages as a result of pacing, for example fewer feelings of frustration and irritability.

I think this is a really important thing, because I think if people can learn it [pacing] early on… then they can keep doing things like work and participating in life I suppose. (Patient 1, 35 years)

Pacing is a big thing. I think psychologically too. If you follow those kind of rules of pacing, you can say to yourself when you're having a bad day, but just remember, I'll probably have a good one tomorrow. (Patient 5, 62 years)

### Theme 5. The Patient Handbook

#### Patient testimonies

The group supported the suggestion of including individual testimonies or accounts from the workshop in the handbook as long as the overall message was positive.

As long as it's positive. We don't want moaning. (Patient 5, 62 years)

They supported the suggestion of film recordings of trial participants included on the website to add relevance and resonance. The youngest participant in the group said a website with video clips of people with long‐term conditions had been very helpful for her when she was first diagnosed.

You need a person to relate to that's been through it [being diagnosed with RA] […] I was like, oh finally someone that understands how you can't go dancing all night anymore at my age. [Laughs] But yeah, I found it really helpful. (Patient 1, 35 years)

She thought that video clips of patients talking about their experience of intensive management would be a valuable resource for others considering it as an option. As with the pictures in the handbook, she recommended video clips of patients from across a broad age range, and the inclusion of both male and female patients.

I think it would be really helpful if patients who have had some degree of intensive treatment […] primarily those on biologics, can talk about how it's really helped them […] it's really scary to go from no medication to lots of medication. (Patient 1, 35 years)

#### Usability of patient handbook

Participants commented on changes which could improve the usability of the handbook. Suggestions included shortening the handbook. This was partly due to overlap of content across chapters but also because it would be physically difficult for an individual with RA who has painful joints to hold and read the heavy handbook. For easier page turning, they suggested a spiral‐bound version.

[…] if you're really sore, a very heavy document is not something you want to be reading. (Patient 5, 62 years)

Some of the scientific content was considered too complex to be included in the handbook. The patients in the group recommended including charts, diagrams and pictures to visually represent the information. They emphasized these would be more helpful to those who are unwell and find it difficult to process the content. Participants liked the bullet points because they focused attention to key points in the text. They agreed that the language was clear but considered references unnecessary.

Pictures […] a lot of the people that you're aiming this at aren't in a very good place […] when I was not well my brain didn't work. […] If I can see it visually, it's much better. (Patient 5, 62 years)

I thought the bullet points were good and I thought the language was quite clear. (Patient 4, 65 years)

Once the trial has been completed, the document will be made publicly available. Patients in the workshop suggested making it available both online and as a hard copy document.

I am totally technophobic, I'm telling you. Doesn't matter how many computer courses you give me, it just doesn't get in there [my head]. (Patient 3, 66 years)

## Discussion

Five main themes from the qualitative data emerged: Rheumatoid Arthritis Treatment, Perceptions of Rheumatoid Arthritis, The Importance of Setting Individualised Goals, Benefits of Self‐Management and the Patient Handbook. Consistent with previous studies,^14^, ^15^ participants requested for complex information to be made more accessible, showed a preference for self‐management information above biomedical information and asked for sections relating to feelings and relationships to be included in the handbook.

Findings from the workshop resulted in important amendments to the content, layout and usability of the second draft of the handbook, which were incorporated into the final version (see Table 2). Any complex scientific information was made more accessible, the length of the handbook was reduced from nine to five chapters (94–43 pages excluding appendices), and the final version was spiral‐bound. Pictures of people spanning a wider diversity of age and ethnicities were added to the handbook, as were sections on ‘talking about your RA’ and ‘how to deal with feelings and relationships’. Practical information that described in detail what taking part in the trial would involve for participants was also included. Relevant anonymous patient testimonies with positive messages from the workshop were inserted to personalize the content of the chapters.

**Table 2:** Changes made to the handbook as a result of the workshop

Accessibility and usability
Increased size of text
Adjusted terminology to be consistent throughout the handbook
Removed references within the text
Added more bullet points
Listed medications in order of most commonly prescribed
Used charts, diagrams and pictures to visually represent information
Made complex scientific information more accessible
Reduced the length of the handbook
Spiral‐bound the final version of the handbook
Content
Clarified the aim and benefits of intensive management
Included information describing what taking part in the trial would involve
Emphasized the flexible and adaptive nature of individual goals
Included pictures of people spanning a wider diversity of age and ethnicities
Added section on ‘talking about your RA’
Added section on ‘how to deal with feelings and relationships’
Added section on ‘dental care’
Included patient testimonies with positive messages

The draft handbook seemed to stimulate in‐depth conversation and descriptions of personal experiences within the group. Participants discussed the social consequences of their condition and the coping strategies they apply to manage their disease. The importance of social support was apparent from the workshop discussions. Participants were keen to increase the knowledge of their partners, friends and family which was reflected by the suggestion that the handbook may be a useful resource for them too. An incidental result of the workshop was that participants had the opportunity to share stories and gain support from each other. These have previously been reported as personal benefits of participating in collaborative research.^21^ As reported in a previous study with RA patients,^22^ social support results in empowering processes such as ‘finding recognition and understanding’ and ‘sharing experiences’.

To our knowledge, this is the first time patients’ views have been integrated into the development of educational materials to support intensive management of patients with RA. Incorporating patients’ views formed one part of this process and also included consultation with expert medical and allied health practitioners and national RA charities (not reported). Strengths of the study include the qualitative workshop methodology which offered insight into the wide range of views that patients had on the topic as well as how they interacted and discussed the issue with other participants.^23^ Patients were consulted throughout the process of the handbook development. This allowed for participant verification, or member checking, which improves the validity of qualitative research findings.^24^ Sending revised versions of the document via email meant that participants were only asked to attend one workshop. This minimized travel and time requirements which have been cited as an adverse effect of engaging patients in research,^25^ and may be particularly relevant for people with RA. Limitations of the study include the participants consisting of all female patients; therefore, no insight was gained into what male patients with RA thought of the handbook. Although RA is predominantly a female disease affecting approximately three times more women than men^26^ gender differences in coping and self‐management styles for patients with RA have previously been reported.^27^ This means providing the same support for men and women with RA may not be effective. Future research involving patients with RA should aim to include both male and female patients.

This study demonstrates that patient contribution to the development of educational material to support intensive management of RA is both feasible and valuable. Future research needs to involve patients both in the process of developing information and in other areas of intervention development. A qualitative evaluation of the use and impact of the handbook with patients and practitioners is planned on completion of the TITRATE trial.

## Conflicts of interest

The authors declare that they have no competing interests.

## Funding

The TITRATE Programme is funded by the National Institute for Health Research's Programme Grants for Applied Research Programme. This is a summary of independent research funded by the National Institute for Health Research (NIHR)'s Programme Grants for Applied Research Programme (Grant Reference Number RP‐PG‐0610‐10066). The views expressed are those of the authors and not necessarily those of the NHS, the NIHR or the Department of Health.
